# Demonstrating antibiotic stewardship while diagnosing and treating bilateral pseudoseptic arthritis: a case report

**DOI:** 10.1186/s13256-024-04568-2

**Published:** 2024-05-10

**Authors:** Rae Tarapore, Sierra Lindsey, Paige Strickland, Robert McKinstry

**Affiliations:** Department of Orthopaedic Surgery, MedStar Union Memorial Hospital 201 E. University Pkwy, Baltimore, MD 21218 USA

**Keywords:** Pseudoseptic arthritis, Case report, Antibiotic stewardship, Septic arthritis

## Abstract

**Introduction:**

Although viscosupplementation is a commonly used treatment for osteoarthritis and is widely regarded as a safe treatment option, it is associated with the rare complication of pseudoseptic arthritis. Most existing case reports that cite this rare complication employed the use of early broad-spectrum antibiotics.

**Case presentation:**

In this case report, we present a 61-year-old African American female patient who presented with bilateral knee pseudoseptic arthritis in the setting of viscosupplementation. She presented 3 days after bilateral viscosupplementation injections with bilateral knee swelling, discomfort, and pain with micromotion. Her white blood cell count (WBC) was 12.83 (4.5–11 normal), her C-reactive protein (CRP) level was 159 mg/L (0–10 normal), and her erythrocyte sedimentation rate (ESR) was 79 mm/hour (0–40 normal). Her left knee aspirate yielded 38,580 WBC with a negative gram stain and negative cultures. Her right knee aspirate yielded 29,670 WBC with a negative gram stain and negative cultures. Through the utilization of careful clinical monitoring, ice therapy, and non-steroidal inflammatory medication, we were able to successfully treat this patient while maintaining proper antibiotic stewardship.

**Conclusion:**

Pseudoseptic arthritis in the setting of viscosupplementation can be adequately treated and monitored without the use of antibiotics.

## Introduction

Viscosupplementation is a commonly used treatment in the arsenal of orthopaedic surgeons when treating osteoarthritis of the knee [4]. Although its clinical effects are the subject of ongoing debate, many surgeons view it as a safe non-operative option which may delay the need for a total knee arthroplasty [4]. This can be especially useful when treating younger patients who have failed other forms of conservative treatment (i.e., physical therapy, non-steroidal anti-inflammatory drugs (NSAIDs), intraarticular corticosteroid injection).

While the viscosupplementation injections are safe options for patients, like any intervention, they come with inherent risks. Pseudoseptic arthritis is a rare complication associated with viscosupplementation, with no occurrence rate currently established in modern literature. In addition, it has been scarcely described in literature via isolated case reports with varying presentations and treatments, most of which have employed broad spectrum antibiotic use [1–3, 6–8]. It is hypothesized that the pseudoseptic arthritis is a hypersensitivity reaction, typically presenting after the second or third injection. In the following case report, we describe what is, to our knowledge, the second reported case of bilateral pseudoseptic that was triggered by viscosupplementation in a patient that had received prior bilateral simultaneous viscosupplementation treatments. We also discuss the subsequent treatment plan that employed appropriate antibiotic stewardship.

### Case

The patient is a 61-year-old African American female with a past medical history only significant for bilateral knee osteoarthritis. Of note, written informed consent was obtained from the patient for publication of this case report. She is an established patient of the senior author and had failed bilateral knee corticosteroid injections, with neither knee receiving significant pain relief. She also had received multiple series of bilateral Hyalgan® (Fidia, Florham Park, NJ) injections in the past with no complications. A series of Hyalgan injections entails a weekly injection for three weeks, resulting in three total injections in each knee per series. The patient had begun a series of Synvisc (Sanofi, Cambridge, MA) injections for which she had tolerated the first two doses bilaterally well. She underwent her third set of Synvisc injections and began having pain bilaterally 12 h after injection.

She presented to the emergency department three days after the third set of injections complaining of bilateral knee swelling and pain, with her left knee being significantly more symptomatic. She had pain and difficulty bearing weight bilaterally, but denied any fever, chills, or malaise on presentation. Her right knee exam was as follows: range of motion from 0–70 degrees secondary to pain, no erythema, mild suprapatellar effusion present, no pain with micromotion. Her left knee exam was as follows: range of motion from 0–30 degrees secondary to pain, no erythema, moderate suprapatellar effusion present, pain with micromotion. Her white blood cell count (WBC) was 12.83 (4.5–11 normal), her C-reactive protein (CRP) level was 159 mg/L (0–10 normal), her erythrocyte sedimentation rate (ESR) was 79 mm/hr (0–40 normal).

Given her clinical presentation, both knees were aspirated. Her left knee aspirate yielded 38,580 WBC with a negative gram stain and negative cultures. Her right knee aspirate yielded 29,670 WBC with a negative gram stain and negative cultures. At this point, her differential diagnosis included pseudoseptic arthritis and septic arthritis. The decision was made to hold antibiotics based off her aspirate results and clinical picture; notably, the treatment team understood that we had a very low threshold to start antibiotics and take the patient to the operating room for arthroscopic irrigation and debridement if her clinical picture worsened. The patient was given non-steroidal anti-inflammatory pain medication (NSAIDs) as well as ice therapy for pain management in house.

**Fig. 1:**
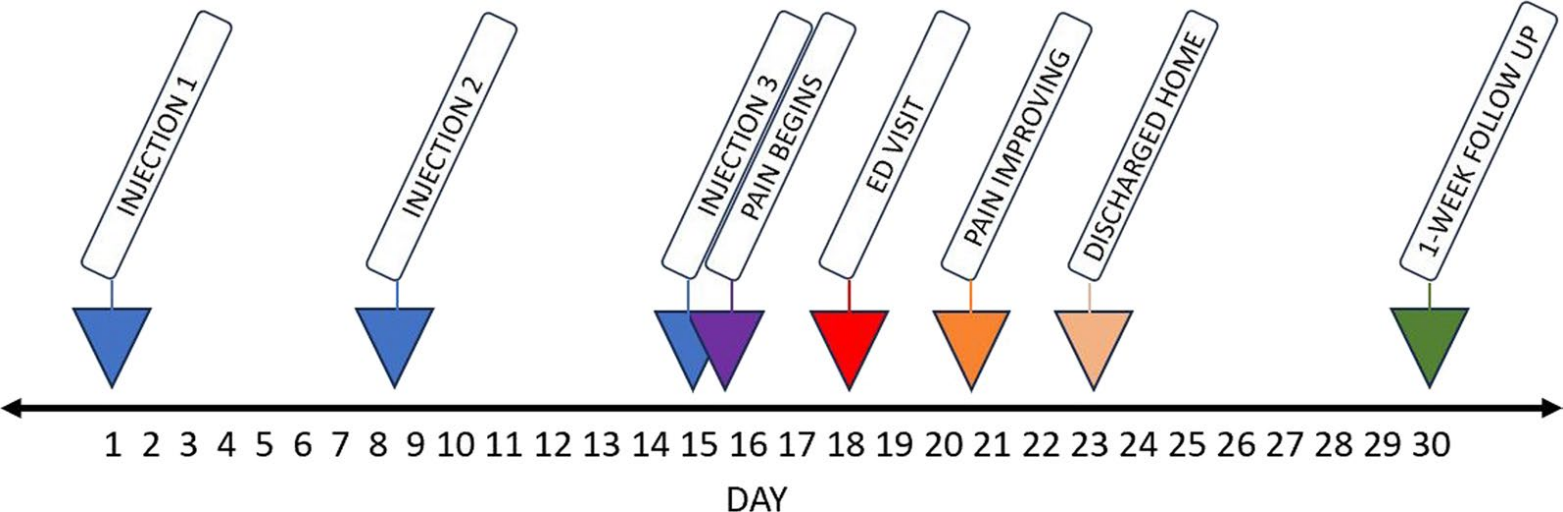
Timeline of events

On hospital day 2, the patient’s left knee pain began to improve with her right knee pain being grossly unchanged. The patient’s management with NSAIDs (ibuprofen 800 mg every 8 h) and physical therapy, as well as strict monitoring of patient’s labs and clinical picture, continued for the next few days. Her pain levels steadily improved day by day in both knees. By her fourth day in the hospital, her CRP had dropped to 87.94 and her WBC had dropped to 7.95. She no longer had any pain with micromotion in her left leg. On hospital day five the patient was discharged with very strict return precautions. At her first clinic visit one week later, the patient reported a complete resolution of her bilateral knee pain to pre-injection levels (Fig. 1).

## Discussion

Treatment of osteoarthritis with viscosupplementation is often characterized as a safe and low risk option for patients who have pain refractory to other conservative treatment options. The most common side effects of viscosupplementation include pain at the injection site, local skin reactions, and joint swelling [4]. Pseudoseptic arthritis as a complication of viscosupplementation presents a unique challenge to clinicians as it closely mimics septic arthritis and requires close monitoring to differentiate between the two pathologies. The danger of misdiagnosing septic arthritis involves risk of damage to articular cartilage that could be mitigated with surgical irrigation and debridement [1, 2].

At this time, the pathophysiology of this clinical sequelae is poorly understood. Theories proposed in the literature include activation of innate immune responses, hypersensitivity to hyaluronic acid, as well as non-specific inflammation in response to viscosupplementation constituents [6]. Furthermore, patient specific immunologic profiles and variations in the formulations of viscosupplementation options may contribute to this pathological sequelae.

Regardless of the pathophysiology, managing this complication appropriately is paramount to patient safety. The differential diagnosis of patients presenting with pseudoseptic arthritis includes crystal induced arthropathy and, crucially, septic arthritis [2, 6]. In the case of monitoring for declaration of septic arthritis versus pseudoseptic arthritis, we recommend admitting patients for monitoring when they demonstrate the signs and symptoms of pseudoseptic arthritis. Such signs and symptoms include a swollen knee that is warm to touch, pain with knee range of motion and possibly pain with micromotion, as well as inability to bear weight.

Distinguishing pseudoseptic arthritis from septic arthritis can be challenging; however, utilization of joint arthrocentesis as a diagnostic tool remains the gold standard for diagnosing septic arthritis [8]. A WBC count of 50 k is classically the accepted cut-off value for diagnosing septic arthritis [8]. The patient in this case report had a left and right knee WBC count of 38 k and 27 k, respectively. Although those are elevated markers, the simultaneous bilateral nature of the symptoms makes diagnosis of native joint septic arthritis less likely. She was kept in the hospital for cultures to grow, to monitor her clinical examination for any potential worsening, and to watch her labs and vitals closely. For this patient, gram stain and cultures remained negative for both knee aspirations throughout her hospitalization and until finalization. In the setting of suspected pseudoseptic arthritis, we recommend initial utilization of NSAIDs and ice therapy both as diagnostic and therapeutic tools. For patients with pseudoseptic arthritis, these treatment options gradually aid in the abatement of symptoms over time by decreasing inflammation. We suggest holding antibiotics to maintain antibiotic stewardship, and more importantly, to not affect the patient’s clinical examination. Any worsening with NSAIDs alone would necessitate progression to antibiotic administration and operative intervention rapidly.

A key aspect of the medical decision making in this case study was the decision to hold antibiotics pending clinical improvement. This differs from much of the existing literature regarding treatment of pseudoseptic arthritis [6–8]. Most similar case reports document treatment with prophylactic intravenous or oral antibiotics. The treating team’s decision to hold anti-infective prophylaxis was largely based off of the patient’s overall clinical picture. On presentation, the patient was hemodynamically stable with no signs of systemic disease. Had she developed a fever, worsening pain, or any vital sign derangements, the patient would have been placed on an antibiotic regimen immediately with plans to perform an arthroscopic irrigation and debridement as soon as possible. Additionally, the patient’s WBC and CRP were taken daily to help correlate with her clinical picture for overall decision making.

The significance of our case study is that to our knowledge, this is only the second case of bilateral pseudoseptic arthritis documented in literature [5]. Furthermore, we demonstrated that this rare complication of viscosupplementation can be appropriately treated without the use of antibiotics with close clinical monitoring. This case may allow for better antibiotic stewardship in the setting of suspected pseudoseptic arthritis and may provide an initial framework for early management in suspected pseudoseptic arthritis. However, we note that it is important to keep patients in the hospital for close monitoring for any deterioration given the detrimental effects of septic arthritis on the joint itself. As patients with pseudoseptic arthritis continue to clinically improve with NSAIDs alone, correlating with improved labs and negative cultures, the decision can be made to continue with non-operative management without antibiotics. Our hope is that the course of this patient’s treatment and the clinical management may prove useful for clinicians who attempt to navigate the treatment options for patients with a similar presentation. This course will allow surgeons to maintain antibiotic stewardship and a clearer picture of any deterioration of the clinical examination that would suggest a septic arthritis.

Our approach to this case was not without limitation. While we employed NSAID and ice therapy, our strategy relied heavily on careful observation. The patient was given strict instructions to notify us if the pain was getting worse. Furthermore, nursing was instructed to be extremely diligent in their care and observation of the patient while in house. Our institution is fortunate enough to have excellent nursing care around the clock with a staff that works primarily in orthopaedic surgery, a luxury not afforded to all institutions. Lastly, there was a dedicated physician or physician assistant (PA) in house at all times to evaluate the patient if necessary. This made our extended observation more feasible, but we understand that this is not the case at other hospitals that may treat pseudoseptic arthritis in the same way we did.

## Conclusion

In this case report, we present an otherwise healthy 61-year-old female who presented with simultaneous pseudoseptic arthritis in both knees in the setting of viscosupplementation. She was admitted for monitoring and both knees were aspirated, yielding WBC counts of 39 k in the left knee and 27 k in the right knee. With ice therapy and NSAID treatment, her inflammatory markers and clinical exam improved daily prior to her uneventful discharge home. This case demonstrates the diagnosis and conservative management of pseudoseptic arthritis in the setting of close inpatient monitoring of the patient while improving upon antibiotic stewardship.

## Data Availability

Not applicable.
